# Nematode mind: exploring the role of the RNA interference pathway in learning, memory and beyond

**DOI:** 10.1098/rstb.2024.0125

**Published:** 2025-06-26

**Authors:** Martyna K. Zwoinska, Varvara Paida, Hwei-yen Chen, Lauren Darkes, Martin I. Lind

**Affiliations:** ^1^ Animal Ecology, Department of Ecology and Genetics, Uppsala Universitet, Uppsala, Sweden; ^2^ Department of Biology, University of Copenhagen, Kobenhavn, Region Hovedstaden, Denmark; ^3^ Biodiversity and Evolution, Department of Biology, Lund University, Lund, Sweden; ^4^ Department of Environmental and Biosciences, Halmstad University, Halmstad, Sweden

**Keywords:** RNA interference (RNAi), *Caenorhabditis elegans*, learning and memory, life-history evolution, NRDE-3 Argonaute protein

## Abstract

*Caenorhabditis* nematodes, particularly the well-known *Caenorhabditis elegans*, have challenged early views of them as hard-wired by demonstrating diverse learning and memory capabilities. This cognitive repertoire, developed with just several hundred neurons, highlights the evolutionary importance of cognitive traits. In this study, we examine the relationship between learning, development, reproduction and lifespan, focusing on its regulation by the conserved RNA interference (RNAi) pathway. Specifically, we examine NRDE-3, a key Argonaute protein in this pathway using an RNAi-defective *nrde-3* mutant to evaluate the effects of this mutation under two diets. Consistent with previous findings, *nrde-3* mutants exhibited a trend towards deteriorated aversive learning; at the same time, no decline in positive learning was observed. Additionally, we found that the *nrde-3* mutant had faster development but also reduced lifespan under the stressful condition of light exposure. We propose that the RNAi pathway, alongside the target of rapamycin and insulin/insulin-like signalling pathways, contributes to the correlated evolution of learning and life-history traits. Notably, recent research has linked the RNAi pathway to the epigenetic inheritance of learned behaviours, presenting new opportunities for integrated investigations into within-generation learning and transgenerational responses. Accordingly, we suggest future research directions that take advantage of the genomic resources, biodiversity and experimental tractability of *Caenorhabditis*.

This article is part of the Theo Murphy meeting issue ‘Selection shapes diverse animal minds’.

## 1. Introduction

### (a) Evolutionary framework to study learning and memory

Within an evolutionary framework, learning—understood as a form of phenotypic plasticity [1]—is one of the adaptive responses to fluctuating selection pressures arising from environmental variability [2–7]. Like other forms of reversible plasticity, learning is favoured in contexts of fine-scale environmental variation, particularly within a single generation, and when conditions are relatively predictable. Beyond environmental factors, fitness costs associated with plastic responses also shape their adaptive value [5], with the costs of learning attracting considerable attention. First, learning incurs exploratory costs inherent in the process of acquiring new information and acting on it. It has also been shown to impose both physiological and evolutionary costs. For example, the processes of learning and memory formation, along with the development and maintenance of the neural machinery supporting cognitive functions, have been linked to delayed development, reduced fertility and shortened lifespan [8–11]. Such negative relationships between learning and life-history traits have often been attributed to resource allocation trade-offs based on the assumption that organisms have a limited pool of resources to divide among competing functions [12,13]. It is important to note, however, that negative correlations between traits can arise for reasons beyond resource allocation trade-offs, including shared developmental or genetic constraints, and correlational selection that favours specific combinations of traits [14–17].

Recent studies on model organisms, such as *Caenorhabditis* nematodes and *Drosophila* fruit flies, have shed light on the functional basis of trait correlations, particularly the roles of conservedmolecular pathways like insulin/insulin-like growth factor 1 (IIS) and mechanistic target of rapamycing (mTOR) [14,16]. These signalling pathways integrate diverse environmental signals, including nutrient availability, photoperiod, immune challenges and other stressors, to coordinate organismal responses. Acting as convergence points, they mediate resource allocation trade-offs while also being shaped by developmental and genetic constraints, as well as correlational selection pressures. Traits inflenced by these pathways include life-history traits, immune responses and cognitive functions, such as learning and memory [14]. More recently, another highly conserved pathway, the RNA interference (RNAi) pathway, has emerged as a potential regulator of both life-history and cognitive traits.

### (b) The role of the RNA interference pathway

Small RNA-mediated gene regulatory pathways, collectively known as RNAi, are fundamental to the regulation of gene expression in both eukaryotes and prokaryotes. These pathways impact nearly every stage of a transcript’s lifecycle, from its synthesis during transcription to its translation, and they are crucial for maintaining genome and transcriptome homeostasis under normal and stressful conditions [18]. The Argonaute (AGO) proteins serve as the key effectors in the RNAi pathways, forming the core of the RNA-induced silencing complex (RISC) alongside small RNAs and various accessory proteins. Mature RISCs bind to complementary messenger RNAs (mRNAs) and silence their expression either through endonucleolytic cleavage (‘slicing’) of the mRNA or by recruiting additional proteins that inhibit translation or promote mRNA degradation [19].

NRDE-3 is a nuclear AGO protein expressed in somatic cells, including neurons, as well as in the germline, where it facilitates the propagation of RNAi responses across generations [18]. Previous research in *Caenorhabditis elegans* has shown that the *nrde-3* RNAi-defective mutant exhibits impaired associative learning [20]. Interestingly, NRDE-3 also appears to influence life-history traits, as germlineless *nrde-3* mutants exhibit a shorter lifespan, a pattern not observed when the germline remains intact [21]. Additionally, components of the RNAi pathway have recently been linked to the epigenetic inheritance of learned behaviours in *C. elegans*, such as the transgenerational avoidance of pathogenic bacteria [22–25]. These findings underscore the potential of the RNAi pathway as a key regulator of gene expression both within and across generations and suggest that RNAi mechanisms may lie at the intersection of plasticity, life-history traits and their correlated evolution.

### (c) *Caenorhabditis* nematodes: simple minds yet profound insights

When the first complete map of the *C. elegans* nervous system was published [26], few believed that these nematodes were capable of learning [27]. However, subsequent research revealed their ability to habituate to repeated stimuli and form short- and long-term associative memories [27–29]. Current forefronts of *Caenorhabditis* research address questions related to the fitness and lifespan consequences of cognitive traits [30–32], different forms and processes underlying memory [29,33] and, more recently, the transgenerational inheritance of epigenetic memory [22–25]. While many findings are mechanistic, research on *Caenorhabditis* is rapidly advancing to incorporate evolutionary and ecological perspectives.

Among *Caenorhabditis* species, *C. elegans*, a workhorse of developmental biology and genetics, has the best-studied ecology [34]. *Caenorhabditis elegans* exhibits a ‘boom and bust’ population dynamic, characterized by rapid growth while feeding on transient bacterial blooms associated with decomposing plant matter, followed by sharp declines as resources dwindle and overcrowding intensifies. The microbial communities that *Caenorhabditis* interact with serve as both food sources and pathogens, exerting strong selection pressures on the cognitive traits of these nematodes. In fact, many learning assays developed for *Caenorhabditis*, including those used in this study, use volatile chemicals—bacterial metabolites that these nematodes may encounter in nature—to assess learning performance [28,29].

### (d) Study aims

Using the experimental tractability of *Caenorhabditis* nematodes (box 1) and building on prior findings linking the RNAi pathway to life-history traits and learning, we investigated the joint regulation of these traits by the key AGO protein NRDE-3. Specifically, we examined how development, reproductive output and lifespan (under late-life light stress) relate to learning performance across appetitive and aversive paradigms. To further explore the ecological context of this regulation, we assessed life-history traits under two distinct food sources, using different bacterial strains. All assays were conducted using the nuclear RNAi-defective *nrde-3* mutant of *C. elegans*. By studying this mutant, we aimed to investigate patterns reflecting the broader properties of the regulatory network, with the assumption that the observed correlations represent the evolutionary history of the RNAi pathway, shaped by both adaptive and non-adaptive forces.

Box 1 **: approaches to study cognitive traits in nematodes**

One of the benefits of using *Caenorhabditis* is their high amenability to diverse research methodologies, ranging from observational and correlational approaches to experimental evolution, and genome editing techniques such as CRISPR-Cas. *Caenorhabditis* nematodes offer several advantages when employing these approaches. For instance, they enable comprehensive phenotypic and life-history assays spanning an entire lifespan of an individual, which facilitates the joint analysis of life-history and cognitive traits. Collectively, these experimental methodologies, coupled with extensive genomic resources and high biodiversity [35,36], make *Caenorhabditis* exceptionally well-suited for testing theoretical models on the evolution and fitness consequences of cognitive traits.

1. Direct measurements of cognitive traits:

— assessment of learning, memory and other cognitive traits in unmanipulated natural or laboratory populations at an individual or population level; and
— can be conducted alongside measurements of other behavioural and life-history traits to examine correlations between cognitive abilities and traits such as development time, reproduction and lifespan [31].

2. Quantitative genetic analysis:

— used to assess components of phenotypic variation in a trait, particularly heritability, which is the proportion of phenotypic variation attributable to genetic variation. This method does not require genomic data or genetic markers; and
— includes the use of parent-offspring regression, existing family data or the creation of pre-defined pedigrees from a single population.

3. Quantitative trait loci mapping:

— correlates genotype and phenotype to identify quantitative trait loci [37];
— genome-wide association studies use whole-genome variant data and can be easily implemented using existing tools linked to extensive collections of natural isolates of *Caenorhabditis* species, such as those available through the *Caenorhabditis* Natural Diversity Resource [35]; and
— linkage mapping: this approach can take advantage of existing recombinant inbred lines and recombinant inbred advanced intercross lines, which are panels of lines derived from crossing two genetically divergent strains [37].

4. Comparative genomics:

— utilizes genomic data from different *Caenorhabditis* species and populations to assess genetic diversity, evolutionary patterns and population structure in relation to cognitive traits; and
— facilitated by extensive genomic databases available for different *Caenorhabditis* species and populations [35,36].

5. Experimental evolution:

— involves controlled manipulations across multiple generations to study evolutionary changes in traits [38];
— includes laboratory-based natural selection (where environmental pressures are manipulated) and artificial selection experiments (where specific traits are selectively bred); and
— particularly powerful if coupled with next-generation sequencing [39].

6. Molecular and genomic manipulations:

— involve the use of gene mutants, cutting-edge techniques like CRISPR-Cas genome editing, as well as genetic manipulations involving gene silencing via RNAi;
— *C. elegans* can ingest environmental RNAs; single guide RNAs can be fed to Cas9-expressing worms to enable genome editing, though injection remains more efficient [40];
— cell- and neuron-specific manipulation of gene expression is possible using cell-specific drivers [41]; and
— essential for understanding gene function and the molecular basis of traits.

7. Epigenetic manipulation:

— the CRISPR-Cas9 system can be repurposed using nuclease-deactivated Cas9 as a sequence-specific, non-mutagenic transcriptional regulator. This allows for precise gene repression (CRISPR interference) or activation (CRISPR activation) [40,42]; and
— although challenging in multicellular organisms, this approach shows promise in decoupling genetic (DNA sequence-based) and epigenetic (non-DNA sequence-based) effects on studied traits [43].

## 2. Material and methods

### (a) Basic maintenance

We used two *C. elegans* genotypes in this study: the nuclear RNAi-defective *nrde-3* (gg66) X (*Caenorhabditis* Genetic Centre name: YY158, hereafter referred to as *nrde-3*(−)), and the wild-type N2 Bristol, which served as a control. Prior to assays, worms were thawed from −80°C storage and propagated for 2–3 generations to eliminate any effects of cryopreservation. To synchronize the populations between generations, we used bleaching, which kills larvae and adults but leaves eggs intact. Unless otherwise specified, worms were maintained on nematode growth medium (NGM) agar plates under standard conditions of 20°C and continuous darkness [44]. Two common *Escherichia coli* strains were used as a food source: the carbenicillin-resistant strain HT115 (L4440), routinely used for RNAi assays, with carbenicillin (1‰) added in the agar, and OP50, the otherwise typical food source for *C. elegans* [44]. These two bacterial strains differ in nutrient composition and result in altered transcriptional and life-history responses in the worms [45].

### (b) Olfactory associative learning assays

*Caenorhabditis elegans* worms exhibit chemotaxis, moving towards or away from specific environmental stimuli, and these innate preferences can be readily modified through conditioning [28,29]. Associative learning paradigms in nematodes often use food or its absence as an unconditioned stimulus, with volatile chemicals such as butanone serving as a conditioned stimulus. During conditioning, the presence or absence of food determines whether the worms’ chemotaxis responses to these chemicals are enhanced or diminished. Successful conditioning results in enhanced responses during appetitive (positive) training, when food is present, or diminished responses during aversive training, when food is absent [28,29]. The butanone conditioning paradigm is well-established for inducing both short- and long-term memories, and its neural correlates have been extensively characterized [46]. Previous research has shown that NRDE-3, expressed in sensory Amphid Wing Neurons C (AWC) neurons, contributes to aversive butanone learning by carrying small RNAs that repress the *odr-1* gene [20]. Interestingly, while AWC neurons detect butanone and, together with interneurons, code short-term memories, the coding of long-term memories induced by butanone is carried out exclusively by the deeper layers of the *C. elegans* neural network [46]. In this study, we used a modified version of previously published protocols for appetitive [47,48] and aversive [20,49] short-term memory. Importantly, while sodium azide was used in previous studies to immobilize worms at the end of chemotaxis assays, we replaced it with refrigeration to eliminate our exposure to this acutely toxic chemical [50]. For the aversive learning assays, we conducted four blocks per genotype (*nrde-3(−*) and N2), with each block comprising four assays: two measuring naïve chemotaxis to butanone and two measuring post-conditioning chemotaxis. For the appetitive learning assays, five such blocks were conducted per worm genotype.

### (c) Aversive learning assay procedure

**Figure 1.**
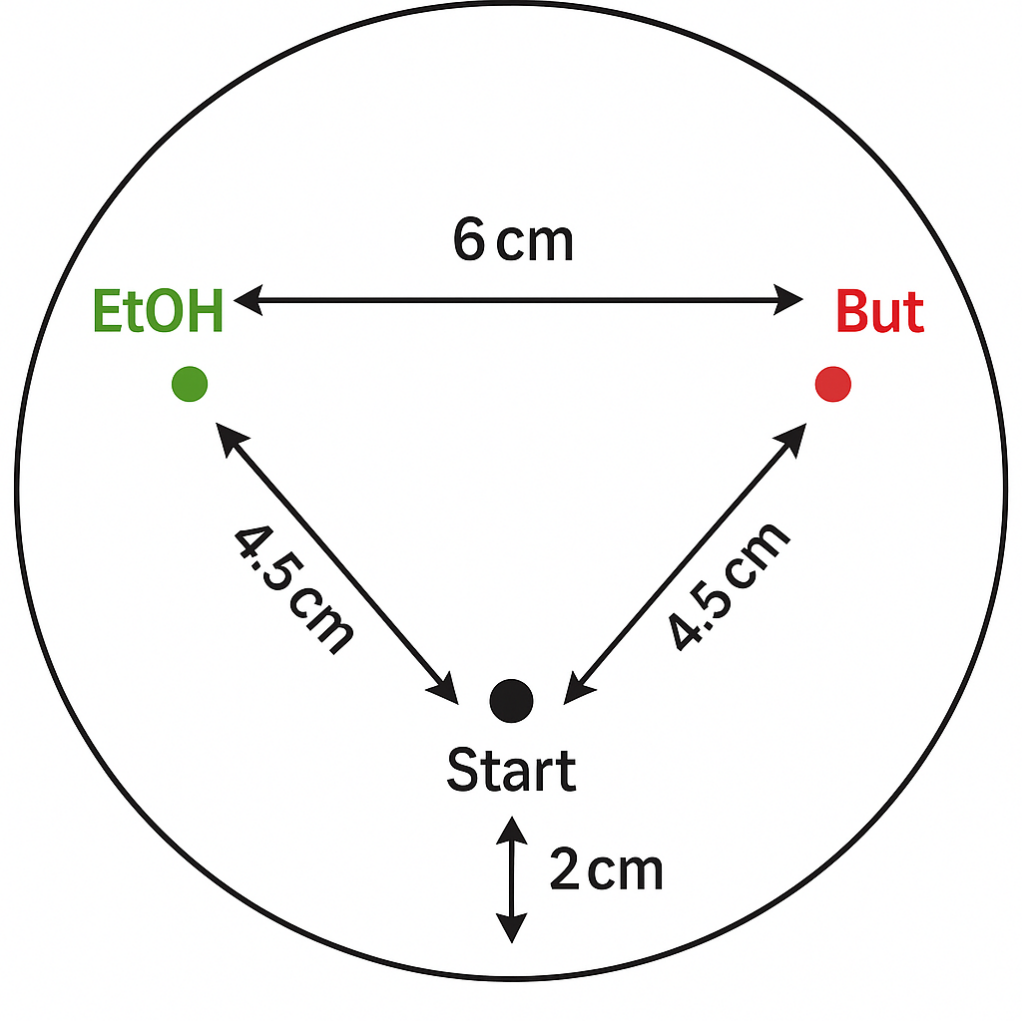
Schematic of the chemotaxis assay plate.

Worms were maintained on 92 mm agar plates seeded with 1 ml of *E. coli* HT115. For each assay, day 3 worms (young adults) were used, with day 0 defined as the day of bleaching; thus, day 3 corresponds to the first reproductive day (i.e. day 1 of adulthood). Worms were collected by washing them off their original plate using standard M9 buffer. The mixture of buffer and worms was transferred to a 10 ml tube. In order to remove any bacterial food from the worm mixture, the worms were washed three times by letting them settle on the bottom of the tube, removing the supernatant, adding fresh buffer and mixing again. For both wild-type and mutant worms, two groups were established: one trained to associate butanone with starvation and assessed for post-conditioning chemotaxis and the other serving as a control to measure naïve chemotaxis to butanone. The trained worms were exposed to 1% butanone (in M9 buffer) for 1 h before measuring their post-conditioning chemotaxis. The naïve chemotaxis groups followed the same procedure, with the only difference being that, instead of being exposed to butanone the worms were exposed to plain buffer for 1 h before the chemotaxis assay. Both naïve and post-conditioning chemotaxis assays were performed on unseeded agar plates with three marked spots on the bottom (figure 1): one spot with ethanol (1 μl), a second spot on the opposite side of the plate with butanone dissolved in 95% ethanol (10%, 1 μl), and a third spot, equidistant from the first two, where the worms were initially placed. After pipetting the worms onto the plate, the plates were inverted and incubated for 1 h. Following incubation, the worms were immobilized by placing the chemotaxis plates in the refrigerator for at least 60 min. Worms within a radius of 3 cm around each spot, as well as the total number of worms on the plate, were counted using a stereoscopic microscope.

To assess the learning ability of the worms, a chemotaxis index (CI) was calculated [51]. The CI was calculated as:


$$CI=\frac{n_{butanone}\;-\;n_{ethanol}}{n_{total}}{\;}_{,}.$$

### (d) Appetitive learning assay procedure

All basic procedures were similar between the aversive and appetitive learning assays, except that in the appetitive learning assays, the trained groups were conditioned to butanone in the presence of food on 60 mm agar plates seeded with 0.5 ml of *E. coli* HT115 and lids streaked with 2 μl of 10% butanone [47,48].

### (e) Development assay

Developmental rate was measured by tracking the percentage of worms reaching adulthood at specific time points. Initially, five hermaphrodite worms were placed on a plate to lay eggs within a 2 h window. Their progeny were then allowed to develop and were scored by counting those with fully formed vulvas, indicating sexual maturity, at three different time points [52]. For the OP50 food source, the time points were 48, 49 and 50 h post egg-laying. For the HT115 food source, the time points were 50, 51 and 52 h owing to logistical reasons. Three replicate plates were used for each time point.

### (f) Reproduction assays

Offspring production for the two nematode strains was measured in two separate assays: one using OP50 as the food source (*n*_N2_ = 30 and *n_nrde-3_* = 28) and another using HT115 (*n*_N2_ = 31 and *n_nrde-3_* = 28). Worms were age-synchronized at the last larval stage (L4) and then were individually placed on seeded NGM agar plates and transferred every 24 h to new plates. Eggs were allowed to hatch and develop for 2 days, after which the larvae were killed by heat shock and counted. We also measured egg size on the OP50 food source (see the electronic supplementary material).

### (g) Lifespan assay

Lifespan was assessed using the same worms previously tested in the reproduction assay. Those that survived to post-reproduction age (day 8 adults) were transferred to an incubator set to an 8 h : 16 h light–dark cycle at approximately 7000 lux, as described in [53]. These conditions created a late-life stressful environment for *C. elegans*. Death was determined by the absence of movement in response to touch and the lack of pharyngeal pumping. The assay was performed with two bacterial strains as food sources (OP50: *n*_N2_ = 32 and *n_nrde-3_* = 29; HT115: *n*_N2_ and *n_nrde-3_* = 30).

### (h) Analyses

All analyses were performed using R 4.1.1 [54], treating worm genotype (N2 or *nrde-3*(−)) as a fixed factor.

Aversive learning performance was analysed using two models, treating the experimental block as either a random or fixed factor. The experimental block is ideally modelled as a random effect when sufficient levels exist to estimate variance reliably in mixed-effect models. However, when this is not feasible, it can be modelled as a fixed effect. In our study, we could estimate the random effect of the experimental block only in the aversive learning assay. For the appetitive learning assay, attempts to fit linear mixed-effects models yielded singular fits, indicating that the random effect structure was unsupported by the data. Consequently, we present models treating the block as a fixed factor for both aversive and appetitive learning assays, alongside a model treating block as a random factor for the aversive learning assay. In all cases, the chemotaxis index of trained worms (post-conditioning chemotaxis) served as the response variable. To account for genotype differences in naïve attraction to butanone, we tested naïve chemotaxis in separate models, with block fitted as either a random or fixed factor, as described above.

Survival in the lifespan assay was analysed using the Cox proportional hazards model. In the development time assay, a binomial regression model was used, with sexual maturity as the response variable. For the reproduction assay, lifetime reproductive success was measured as the total number of offspring produced by each individual. We also calculated rate-sensitive fitness (*λ*_ind_), which uses both the number and the timing of offspring and is equivalent to the intrinsic rate of population growth. First, the population projection matrix was constructed per individual plate, and subsequently, the dominant eigenvalue from the matrix was calculated, following [55]. All non-natural mortality events, such as accidental killing or loss during transfer, were excluded from the analysis. To account for this, survival was set to 1 in the projection matrix. In this case, *λ* serves as a reliable proxy for fitness in worm populations, as it places greater weight on early reproduction.

## 3. Results

### (a) Impact of *nrde-3* mutation on learning performance

**Figure 2.**
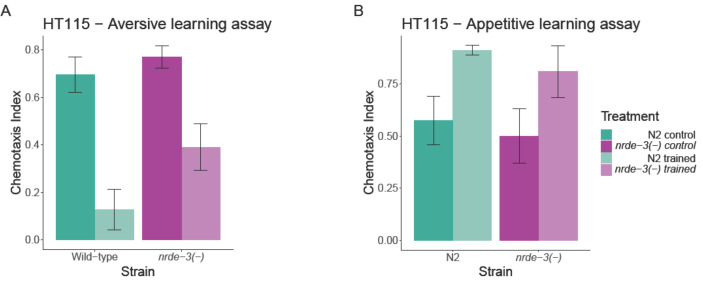
Effects of *nrde-3* mutation on learning. Opaque colours represent naïve, while transparent colours represent trained worms. Bars represent the chemotaxis index of strains of the indicated group (mean ± s.e.). (A) Aversive learning assay. (B) Appetitive learning assay.

Given that *nrde-3*(−) mutants have previously shown impaired aversive learning under the HB101 diet [20], we aimed to determine whether this pattern persists with a different food source (HT115). In the aversive learning assay (associating a smell with the absence of food), the wild-type exhibited a significantly lower post-conditioning chemotaxis index (indicating better learning) than the *nrde-3(*−) mutant when the experimental block was treated as a random factor in the model (table 1; figure 2A). When the experimental block was instead treated as a fixed factor, the effect remained quantitatively similar but did not reach statistical significance (*F*_1,13_ = 3.7979, *p* = 0.073). Importantly, no difference in the naïve (pre-conditioning) chemotaxis index was observed between the two genotypes (block as a random factor: table 1; as a fixed factor: *F*_1,13_ = 1.0192, *p* = 0.331).

**Table 1.** Linear mixed-effects model summaries for aversive learning assays: post-conditioning (post-learning) and naïve chemotaxis indices (CI).

aversive learning linear mixed-effects models									
post-conditioning CI					naïve CI				
fixed effects	estimate	s.e.	*χ*^2^-value	*p*‐value	fixed effects	estimate	s.e.	*χ*^2^-value	*p*‐value
intercept	0.1271	0.1132	1.261	0.262	intercept	0.6952	0.0816	72.669	<0.001
genotype *nrde-3*	0.2627	0.1017	6.675	0.0098	genotype *nrde-3*	0.0743	0.0570	1.700	0.1924
**random effects**	**variance**	**s.d.**		**random effects**	**variance**		**s.d.**		
block (Intercept)	0.03059	0.1749		block (Intercept)	0.0201		0.1418		
residual	0.04136	0.2034		residual	0.0130		0.1140		

In the appetitive learning assay (associating a smell with food), no significant differences were found between the genotypes for either trained worms (*F*_1,17_ = 0.6544, *p* = 0.430) or naïve worms (*F*_1,17_ = 0.2813, *p* = 0.603; figure 2B).

### (b) Impact of *nrde-3* on development

**Figure 3.**
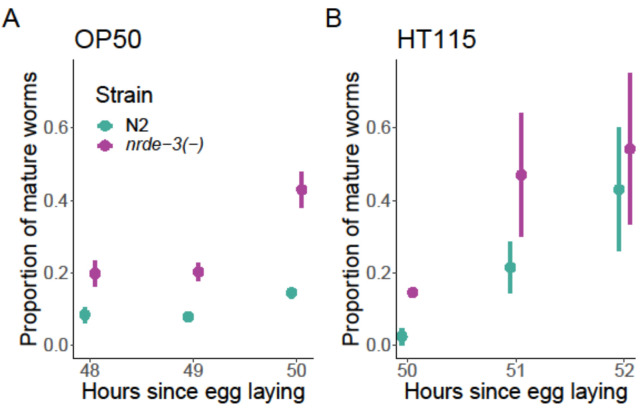
Effects of *nrde-3* mutation on development time. Development time expressed as a proportion of developed worms calculated as mature/total in each plate. Three different time points were used. (A) Proportion of mature *E. coli* OP50 fed worms. (B) Proportion of mature *E. coli* HT115 (L4440) fed worms.

*Nrde-3*(−) worms developed faster than the N2 wild-type worms on both OP50 ($\chi_{1}^{2}$ = 18.2318, *p* < 0.001; figure 3A) and HT115 ($\chi_{1}^{2}$ = 9.3526, *p* < 0.001; figure 3B). The proportion of mature worms increased over time for both food sources (OP50: $\chi_{1}^{2}$ = 9.0496, *p* = 0.003; HT115: $\chi_{1}^{2}$ = 21.5602, *p* < 0.001), with the *nrde-3*(−) consistently showing a higher proportion of mature individuals.

### (c) Impact of *nrde-3* on reproduction

**Figure 4.**
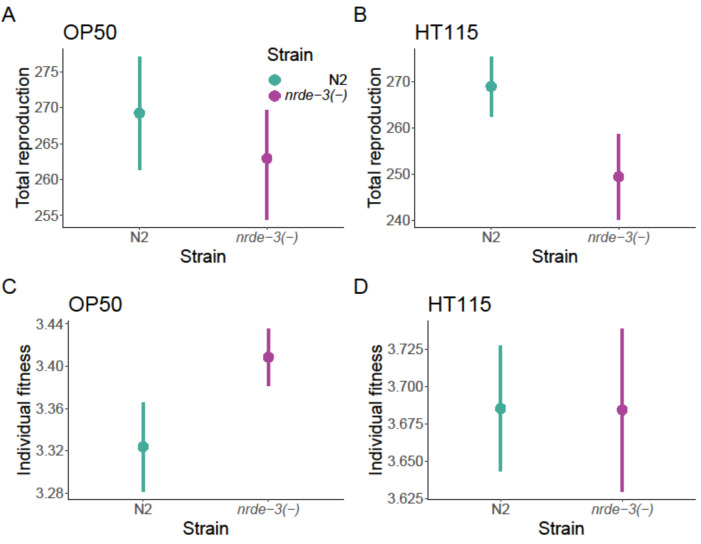
Effects of *nrde-3* mutation on reproduction. (A) Lifetime reproductive success of *E. coli* OP50 fed worms, reproduction mean points with ±s.e. bars. (B) Lifetime reproductive success of *E. coli* HT115 (L4440) fed worms, reproduction mean points with ±s.e. bars. (C) Individual fitness (*λ*_ind_) calculated using the Euler–Lotka equation for *E. coli* OP50 fed worms, median with 95% confidence intervals is displayed. (D) Individual fitness (*λ*_ind_) calculated using the Euler–Lotka equation for *E. coli* HT115 (L4440) fed worms, median with 95% confidence intervals is displayed.

*Nrde-3*(−) worms had a lower total reproductive output when OP50 was used as a food source (*F*_1,57_ = 6.42, *p* = 0.014; figure 4A), but there was no significant difference in rate-sensitive individual fitness (*λ*_ind_) between the strains (*F*_1,57_ = 0.966, *p* = 0.330; figure 4B). However, when HT115 was used, the *nrde-3*(−) mutants produced a similar number of offspring as the N2 wild-type (*F*_1,56_ = 0.0098, *p* = 0.921; figure 4C) and showed a trend towards higher *λ*_ind_ (*F*_1,56_ = 2.849, *p* = 0.097; figure 4D). No differences in egg size were observed between the strains (*F*_1,34_ = 0.1842, *p* = 0.670; electronic supplementary material, figure S2).

### (d) Impact of *nrde-3* on lifespan

**Figure 5.**
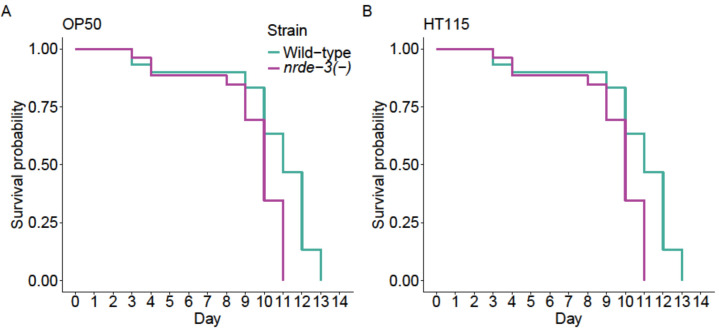
Impact of *nrde-3* mutation on lifespan, represented by survival probability. In both cases, there was light during incubation (8 L : 16 D) as a late-life stressor. (A) Survival probability when feeding on *E. coli* OP50. (B) Survival probability when feeding on *E. coli* HT115 (L4440).

The mutants had shorter lifespans than the N2 wild-type, both when fed OP50 (*z*_1_ = 3.598, *p* < 0.001; figure 5A) and HT115 (*z*_1_ = 3.65, *p* < 0.001; figure 5B).

## 4. Discussion

We investigated the RNAi pathway as a potential functional underpinning of the correlated evolution between learning and life-history traits. Using a loss-of-function mutant of the nuclear NRDE-3 protein, a key RNAi component, we confirmed that these mutants, previously known for impaired aversive learning on a single food source, exhibit similar deficits on another food source. Surprisingly, we did not observe a learning deficit in appetitive learning, suggesting that NRDE-3 may play little role in this context. We also demonstrated that *nrde-3*(−) worms develop faster and, under light stress, live shorter lives. The mutation did not incur a cost in rate-sensitive individual fitness—probably the most relevant measure given nematodes’ boom-and-bust population dynamics—and had either no or a negative impact on total reproductive output, depending on the food source.

*Nrde-3*(−) mutants reached sexual maturity faster than their N2 wild-type counterparts; however, this advantage came at the cost of a reduced lifespan under the stress of late-life light exposure. Previous studies have found a reduction in lifespan in germlineless *nrde-3*(−) worms, but not when the germline was intact [21]. Our finding of the lifespan cost associated with the *nrde-3* mutation in intact worms under stressful conditions supports the broader observation that life-history trade-offs become more pronounced when organisms face stress [56]. The role of *nrde-3* in development has not previously been reported, but our findings align with the observation of an evolutionary trade-off between development and lifespan [57,58]. Additionally, our finding of either no significant effect or a trend towards increased rate-sensitive individual fitness in *nrde-3*(−) mutants, depending on the food source, suggests that downregulating *nrde-3* may not incur fitness costs, at least when food is readily available as in our experimental assays. It should be noted that using rate-sensitive fitness as the fitness measure benefits the *nrde-3* mutant on both OP50 (mutants have lower total reproduction but similar rate-sensitive fitness to the wild-type) and HT115 (no differences in total reproduction, but mutants have higher rate-sensitive fitness).

Our results also demonstrate an aversive learning deficit in *nrde-3* mutants, consistent with previous reports [20]. When the experimental block was treated as a random factor in the model (as recommended when the variability owing to experimental blocks can be estimated), the differences in post-conditioning chemotaxis to butanone between N2 control worms and *nrde-3*(−) were statistically significant. However, these differences were marginally non-significant when the block was instead modelled as a fixed effect. It is also important to note differences in our experimental protocol compared to previous studies. Most prior research, including the studies on which our learning protocols were based [20,47–49], used sodium azide to immobilize worms at odourant spots during chemotaxis assays. Owing to its acute toxicity to humans [50], we opted for refrigeration, capturing worms present at the odourant spot after 1 h, rather than all worms that passed the spot during the assay. This method immobilizes a subset of the worms immobilized by sodium azide. Despite not achieving statistical significance in one of the analyses, the overall pattern in our data aligns with previously reported learning deficits [20]. Finally, while the role of NRDE-3 in aversive learning has been studied previously, its role in appetitive learning remains unknown. Interestingly, our appetitive learning assay showed no tendency towards decreased learning in *nrde-3*(−) mutants. This highlights the potential for NRDE-3 to play distinct roles across different learning paradigms, which warrants further investigation.

While the contribution of specific processes driving the patterns of correlations between traits—such as those between development and lifespan or learning and other life-history traits—remains complex and difficult to quantify, these correlations are often interpreted within the framework of evolved life-history strategies. A life-history strategy encompasses a suite of co-evolving traits that respond collectively to selective pressures while being simultaneously shaped by trade-offs and constraints [12,13]. Traditionally, the fast-slow continuum has been a central axis for understanding variation in life-history strategies. Organisms at the fast end of the continuum develop rapidly, reach reproductive maturity early, and have shorter lifespans. By contrast, organisms at the slow end exhibit slower development, delayed reproduction and longer lifespans. However, recent research suggests that this continuum is just one of several axes structuring life-history variation, underscoring the complexity of evolutionary pathways that shape trait relationships [59]. In our study, we assume that the correlations between traits we observed have been shaped by evolutionary forces, although we cannot determine the specific forces involved. We note, however, that the pattern of changes in life-history traits mediated by *nrde-3* loss-of-function broadly aligns with a shift towards a faster life-history strategy.

Finally, while most studies on *C. elegans* use only one food source, *C. elegans* naturally live in complex environments where they encounter diverse microbial communities [34]. Different bacterial diets vary in nutrient composition, leading to distinct physiological and metabolic responses, including variations in gene expression [45]. Our aim was not to directly compare the two food sources; therefore, they were tested in separate assays that cannot be formally compared statistically. Rather, we focused on investigating the effect of *nrde-3*(−) across these distinct dietary conditions. Notably, our results indicate that the life-history effects of *nrde-3*(−) were relatively consistent across the two diets, suggesting a repeatable pattern despite the differing nutrient compositions.

## 5. Conclusions and future directions

Our study shows that the RNAi-defective *nrde-3* mutant of *C. elegans* exhibits accelerated development, a shortened lifespan under late-life stress, and, consistent with an earlier study, a trend towards impaired aversive associative learning. Complementing research on conserved IIS and mTOR pathways, our results suggest that the RNAi pathway may also play a role in regulating life-history and cognitive traits, potentially mediating their correlated evolution. Future studies should explore whether variation in *nrde-3*-associated pathways and other RNAi components drives differences in learning and life-history traits across *Caenorhabditis* species.

### (a) RNA interference pathway at the crossroads of life history, cognition and transgenerational memory inheritance

In addition to the increasingly well-understood role of different components of the RNAi pathway in within-generation learning and memory, recent studies have also highlighted its involvement in transgenerational memory inheritance. Transgenerational memory describes the epigenetic transmission of learnt behaviours across generations. For example, *Caenorhabditis* worms are naturally attracted to various *Pseudomonas* bacteria [22,25,60] but learn to avoid these pathogens after illness and pass this learnt avoidance to their offspring across several generations [22–25]. This behaviour is mediated by epigenetic mechanisms involving small RNAs and histone modifications [23].

Evidence suggests that the molecular mechanisms underlying transgenerational memory differ from those of long-term memory, although both rely on RNAi machinery, with RNAi components influencing both processes simultaneously [20,23,61–64]. For example, small RNAs at synapses may regulate gene expression during memory consolidation [63,64], while neuronal small RNAs transported to the germline [65] may mediate transgenerational inheritance [66]. Notably, RNAi-defective *nrde-3* mutants exhibit both impaired transgenerational epigenetic inheritance [60] and deficits in aversive learning [20]. Further research is needed to characterize the shared and distinct regulatory roles of RNAi pathway components in transgenerational memory and other memory processes, as well as their impact on life-history traits.

Addressing these complex relationships requires integrating diverse research methodologies. *Caenorhabditis* species are already known to differ in their RNAi effector repertoires [66] and RNAi response capabilities [67]. Moreover, they exhibit variation in learning and transgenerational memory across the genus [60]. These differences raise intriguing questions about how learning, memory and their underlying processes evolve under different selective pressures. Given their experimental tractability (box 1), extensive genomic resources and remarkable biodiversity, we argue that *Caenorhabditis* nematodes remain an under-used system in the study of cognitive ecology.

## Data Availability

Phenotypic data from the assays, as well as the R scripts used for their analyses, can be found at [68]. Supplementary material is available online [69].

## References

[1] Wright J, Haaland TR, Dingemanse NJ, Westneat DF. 2022 A reaction norm framework for the evolution of learning: how cumulative experience shapes phenotypic plasticity. Biol. Rev. **97**, 1999–2021. (10.1111/brv.12879) 35790067 PMC9543233

[2] Kozielska M, Weissing FJ. 2024 A neural network model for the evolution of learning in changing environments. PLoS Comput. Biol. **20**, e1011840. (10.1371/journal.pcbi.1011840) 38289971 PMC10857588

[3] Dunlap AS, Stephens DW. 2016 Reliability, uncertainty, and costs in the evolution of animal learning. Curr. Opin. Behav. Sci. **12**, 73–79. (10.1016/j.cobeha.2016.09.010)

[4] Botero CA, Weissing FJ, Wright J, Rubenstein DR. 2015 Evolutionary tipping points in the capacity to adapt to environmental change. Proc. Natl Acad. Sci. USA **112**, 184–189. (10.1073/pnas.1408589111) 25422451 PMC4291647

[5] Kronholm I. 2022 Evolution of anticipatory effects mediated by epigenetic changes. Environ. Epigenetics **8**, c007. (10.1093/eep/dvac007) PMC9031056 35475265

[6] Vinton AC, Gascoigne SJL, Sepil I, Salguero-Gómez R. 2022 Plasticity’s role in adaptive evolution depends on environmental change components. Trends Ecol. Evol. **37**, 1067–1078. (10.1016/j.tree.2022.08.008) 36153155

[7] Dunlap AS, Austin MW, Figueiredo A. 2019 Components of change and the evolution of learning in theory and experiment. Anim. Behav. **147**, 157–166. (10.1016/j.anbehav.2018.05.024)

[8] Burger JMS, Kolss M, Pont J, Kawecki TJ. 2008 Learning ability and longevity: a symmetrical evolutionary trade-off in Drosophila. Evolution **62**, 1294–1304. (10.1111/j.1558-5646.2008.00376.x) 18363867

[9] Mery F, Kawecki TJ. 2004 An operating cost of learning in Drosophila melanogaster. Anim. Behav. **68**, 589–598. (10.1016/j.anbehav.2003.12.005)

[10] Mery F, Kawecki TJ. 2003 A fitness cost of learning ability in Drosophila melanogaster. Proc. R. Soc. B **270**, 2465–2469. (10.1098/rspb.2003.2548) PMC1691529 14667336

[11] Snell-Rood EC, Davidowitz G, Papaj DR. 2011 Reproductive tradeoffs of learning in a butterfly. Behav. Ecol. **22**, 291–302. (10.1093/beheco/arq169)

[12] Stearns S. 1998 The evolution of life histories. Oxford, UK: Oxford University Press. (10.1093/oso/9780198577416.001.0001)

[13] Roff D. 2002 Life history evolution. Sunderland, MA: Sinauer Associates. (10.1016/B978-0-12-384719-5.00087-3)

[14] Regan JC, Froy H, Walling CA, Moatt JP, Nussey DH. 2020 Dietary restriction and insulin‐like signalling pathways as adaptive plasticity: a synthesis and re‐evaluation. Funct. Ecol. **34**, 107–128. (10.1111/1365-2435.13418)

[15] Agrawal AA. 2020 A scale‐dependent framework for trade‐offs, syndromes, and specialization in organismal biology. Ecology **101**, 2924. (10.1002/ecy.2924) 31660584

[16] Flatt T. 2020 Life-history evolution and the genetics of fitness components in Drosophila melanogaster. Genetics **214**, 3–48. (10.1534/genetics.119.300160) 31907300 PMC6944413

[17] Lind MI, Carlsson H, Duxbury EML, Ivimey-Cook E, Maklakov AA. 2021 Cost-free lifespan extension via optimization of gene expression in adulthood aligns with the developmental theory of ageing. Proc. R. Soc. B **288**, 20201728. (10.1098/rspb.2020.1728) PMC7893226 33529563

[18] Seroussi U *et al*. 2023 A comprehensive survey of C. elegans argonaute proteins reveals organism-wide gene regulatory networks and functions. eLife **12**, e83853. (10.7554/elife.83853) 36790166 PMC10101689

[19] Iwakawa H oki, Tomari Y. 2022 Life of RISC: formation, action, and degradation of RNA-induced silencing complex. Mol. Cell **82**, 30–43. (10.1016/j.molcel.2021.11.026) 34942118

[20] Juang BT, Gu C, Starnes L, Palladino F, Goga A, Kennedy S, L’Etoile ND. 2013 Endogenous nuclear RNAi mediates behavioral adaptation to odor. Cell **154**, 1010–1022. (10.1016/j.cell.2013.08.006) 23993094 PMC4274153

[21] Cohen-Berkman M, Dudkevich R, Ben-Hamo S, Fishman A, Salzberg Y, Waldman Ben-Asher H, Lamm AT, Henis-Korenblit S. 2020 Endogenous siRNAs promote proteostasis and longevity in germline-less Caenorhabditis elegans. eLife **9**, e50896. (10.7554/elife.50896) 32213289 PMC7136021

[22] Sengupta T, St Ange J, Moore R, Kaletsky R, Marogi J, Myhrvold C, Gitai Z, Murphy CT. 2023 A natural bacterial pathogen of C. elegans uses a small RNA to induce transgenerational inheritance of learned avoidance. BioRxiv 07.20.549962. (10.1101/2023.07.20.549962) PMC10977744 38547071

[23] Moore RS, Kaletsky R, Murphy CT. 2021 Protocol for transgenerational learned pathogen avoidance behavior assays in Caenorhabditis elegans. STAR Protoc. **2**, 100384. (10.1016/j.xpro.2021.100384) 33748786 PMC7960678

[24] Kaletsky R, Moore R, Sengupta T, Seto R, Ceballos-Llera B, Murphy CT. 2024 Absence of evidence is not evidence of absence: the many flaws in the case against transgenerational epigenetic inheritance of pathogen avoidance in C. elegans. Genetics. (10.1101/2024.06.07.597568) PMC12080996 40372780

[25] Seto R, Brown R, Kaletsky R, Parsons LR, Moore RS, Murphy CT. 2024 Pseudomonas fluorescens 15 small RNA Pfs1 mediates transgenerational epigenetic inheritance of pathogen avoidance in C. elegans through the Ephrin receptor VAB-1. bioRxiv 2024.05.23.595334. (10.1101/2024.05.23.595334)

[26] White J, Southgate E, Thomson J, Brenner S. 1986 The structure of the nervous system of the nematode Caenorhabditis elegans. Phil. Trans. R. Soc. Lond. B **314**, 1–340. (10.1098/rstb.1986.0056) 22462104

[27] Rankin C. 2020 But can they learn? My accidental discovery of learning and memory in C. elegans. J. Neurogenetics **34**, 251–254. (10.1080/01677063.2020.1833009) 33446016

[28] Wong JSH, Rankin CH. 2019 *Caenorhabditis elegans* learning and memory. In Oxford research encyclopedia of neuroscience. Oxford, UK: Oxford University Press. (10.1093/acrefore/9780190264086.013.282)

[29] Yu AJ, Rankin CH. 2022 Learning and memory in the nematode *Caenorhabditis elegans*. In Evolution of learning and memory mechanisms (eds KL Hollis, MA Krause, MR Papini), pp. 15–32. Cambridge, UK: Cambridge University Press. (10.1017/9781108768450.004)

[30] Stein GM, Murphy CT. 2012 The Intersection of aging, longevity pathways, and learning and memory in C. elegans. Front. Genet. **3**, 259. (10.3389/fgene.2012.00259) 23226155 PMC3509946

[31] Zwoinska MK, Kolm N, Maklakov AA. 2013 Sex differences in cognitive ageing: testing predictions derived from life-history theory in a dioecious nematode. Exp. Gerontol. **48**, 1469–1472. (10.1016/j.exger.2013.09.008) 24120565

[32] Zwoinska MK, Lind MI, Cortazar-Chinarro M, Ramsden M, Maklakov AA. 2016 Selection on learning performance results in the correlated evolution of sexual dimorphism in life history. Evolution **70**, 342–357. (10.1111/evo.12862) 26787139

[33] Rahmani A, Chew YL. 2021 Investigating the molecular mechanisms of learning and memory using Caenorhabditis elegans. J. Neurochem. **159**, 417–451. (10.1111/jnc.15510) 34528252

[34] Schulenburg H, Félix MA. 2017 The natural biotic environment of Caenorhabditis elegans. Genetics **206**, 55–86. (10.1534/genetics.116.195511) 28476862 PMC5419493

[35] Crombie TA *et al*. 2024 CaeNDR, the Caenorhabditis natural diversity resource. Nucleic Acids Res. **52**, D850–D858. (10.1093/nar/gkad887) 37855690 PMC10767927

[36] Harris TW *et al*. 2020 WormBase: a modern model organism information resource. Nucleic Acids Res. **48**, D762–D767. (10.1093/nar/gkz920) 31642470 PMC7145598

[37] Andersen EC, Rockman MV. 2022 Natural genetic variation as a tool for discovery in Caenorhabditis nematodes. Genetics **220**, b156. (10.1093/genetics/iyab156) PMC8733454 35134197

[38] Teotónio H, Estes S, Phillips PC, Baer CF. 2017 Experimental evolution with Caenorhabditis nematodes. Genetics **206**, 691–716. (10.1534/genetics.115.186288) 28592504 PMC5499180

[39] Schlötterer C, Kofler R, Versace E, Tobler R, Franssen SU. 2015 Combining experimental evolution with next-generation sequencing: a powerful tool to study adaptation from standing genetic variation. Heredity **114**, 431–440. (10.1038/hdy.2014.86) 25269380 PMC4815507

[40] Kim HM, Hong Y, Chen J. 2022 A decade of CRISPR-Cas genome editing in C. elegans. Int. J. Mol. Sci. **23**, 15863. (10.3390/ijms232415863) 36555505 PMC9781986

[41] Nava S *et al*. 2023 A cGAL-UAS bipartite expression toolkit for Caenorhabditis elegans sensory neurons. Proc. Natl Acad. Sci. USA **120**, e2221680120. (10.1073/pnas.2221680120) 38096407 PMC10743456

[42] Dominguez AA, Lim WA, Qi LS. 2016 Beyond editing: repurposing CRISPR–Cas9 for precision genome regulation and interrogation. Nat. Rev. Mol. Cell Biol. **17**, 5–15. (10.1038/nrm.2015.2) 26670017 PMC4922510

[43] Fischer F, Benner C, Goyala A, Grigolon G, Vitiello D, Wu J, Zarse K, Ewald CY, Ristow M. 2022 Ingestion of single guide RNAs induces gene overexpression and extends lifespan in Caenorhabditis elegans via CRISPR activation. J. Biol. Chem. **298**, 102085. (10.1016/j.jbc.2022.102085) 35636511 PMC9243178

[44] Stiernagle T. 2006 Maintenance of C. elegans. WormBook 1–11. (10.1895/wormbook.1.101.1) PMC4781397 18050451

[45] Stuhr NL, Curran SP. 2020 Bacterial diets differentially alter lifespan and healthspan trajectories in C. elegans. Commun. Biol. **3**, 653. (10.1038/s42003-020-01379-1) 33159120 PMC7648844

[46] Pritz C, Itskovits E, Bokman E, Ruach R, Gritsenko V, Nelken T, Menasherof M, Azulay A, Zaslaver A. 2023 Principles for coding associative memories in a compact neural network. eLife **12**, e74434. (10.7554/elife.74434) 37140557 PMC10159626

[47] Kauffman AL, Ashraf JM, Corces-Zimmerman MR, Landis JN, Murphy CT. 2010 Insulin signaling and dietary restriction differentially influence the decline of learning and memory with age. PLoS Biol. **8**, e1000372. (10.1371/journal.pbio.1000372) 20502519 PMC2872642

[48] Kauffman A, Parsons L, Stein G, Wills A, Kaletsky R, Murphy C. 2011 C. elegans positive butanone learning, short-term, and long-term associative memory assays. J. Vis. Exp. 2490. (10.3791/2490) 21445035 PMC3197297

[49] Colbert HA, Bargmann CI. 1995 Odorant-specific adaptation pathways generate olfactory plasticity in C. elegans. Neuron **14**, 803–812. (10.1016/0896-6273(95)90224-4) 7718242

[50] Frederick KA, Babish JG. 1982 Evaluation of mutagenicity and other adverse effects of occupational exposure to sodium azide. Regul. Toxicol. Pharmacol. **2**, 308–322. (10.1016/0273-2300(82)90004-6) 7185097

[51] Torayama I, Ishihara T, Katsura I. 2007 Caenorhabditis elegans integrates the signals of butanone and food to enhance chemotaxis to butanone. J. Neurosci. **27**, 741–750. (10.1523/jneurosci.4312-06.2007) 17251413 PMC6672901

[52] Travers LM, Carlsson H, Lind MI, Maklakov AA. 2021 Beneficial cumulative effects of old parental age on offspring fitness. Proc. R. Soc. B **288**, 20211843. (10.1098/rspb.2021.1843) PMC8511764 34641727

[53] Ivimey-Cook ER, Sales K, Carlsson H, Immler S, Chapman T, Maklakov AA. 2021 Transgenerational fitness effects of lifespan extension by dietary restriction in Caenorhabditis elegans. Proc. R. Soc. B **288**, 20210701. (10.1098/rspb.2021.0701) PMC8113902 33975472

[54] R Core Team. 2021 R: a language and environment for statistical computing. Vienna, Austria: R Foundation for Statistical Computing.

[55] McGraw JB, Caswell H. 1996 Estimation of individual fitness from life-history data. Am. Nat. **147**, 47–64. (10.1086/285839)

[56] Speakman JR *et al*. 2015 Oxidative stress and life histories: unresolved issues and current needs. Ecol. Evol. **5**, 5745–5757. (10.1002/ece3.1790) 26811750 PMC4717350

[57] Lind MI, Chen H, Meurling S, Guevara Gil AC, Carlsson H, Zwoinska MK, Andersson J, Larva T, Maklakov AA. 2017 Slow development as an evolutionary cost of long life. Funct. Ecol. **31**, 1252–1261. (10.1111/1365-2435.12840)

[58] Janssens L, Stoks R. 2018 Rapid larval development under time stress reduces adult life span through increasing oxidative damage. Funct. Ecol. **32**, 1036–1045. (10.1111/1365-2435.13068)

[59] Stott I *et al*. 2024 Life histories are not just fast or slow. Trends Ecol. Evol. **39**, 830–840. (10.1016/j.tree.2024.06.001) 39003192

[60] Burton NO, Burkhart KB, Kennedy S. 2011 Nuclear RNAi maintains heritable gene silencing in Caenorhabditis elegans. Proc. Natl Acad. Sci. USA **108**, 19683–19688. (10.1073/pnas.1113310108) 22106253 PMC3241819

[61] Fitz-James MH, Cavalli G. 2022 Molecular mechanisms of transgenerational epigenetic inheritance. Nat. Rev. Genet. **23**, 325–341. (10.1038/s41576-021-00438-5) 34983971 PMC7619059

[62] Miska EA, Rechavi O. 2021 Can brain activity transmit transgenerationally? Curr. Topics Dev. Biol **144**, 1–18. (10.1016/bs.ctdb.2021.03.001) 33992150

[63] Ortega-de San Luis C, Ryan TJ. 2022 Understanding the physical basis of memory: molecular mechanisms of the engram. J. Biol. Chem. **298**, 101866. (10.1016/j.jbc.2022.101866) 35346687 PMC9065729

[64] Levenson JM, Sweatt JD. 2005 Epigenetic mechanisms in memory formation. Nat. Rev. Neurosci. **6**, 108–118. (10.1038/nrn1604) 15654323

[65] Wong C, Jurczak EM, Roy R. 2024 Neuronal exosomes transport an miRISC cargo to preserve stem cell integrity during energy stress. Cell Rep. **43**, 114851. (10.1016/j.celrep.2024.114851) 39392750

[66] Dalzell J. 2011 RNAi effector diversity in nematodes. PLoS Neglected Trop. Dis. **5**, e1176. (10.1371/journal.pntd.0001176) PMC3110158 21666793

[67] Nuez I, Félix MA. 2012 Evolution of susceptibility to ingested double-stranded RNAs in Caenorhabditis nematodes. PLoS ONE **7**, e29811. (10.1371/journal.pone.0029811) 22253787 PMC3256175

[68] Zwoinska M, Paida V, Chen HY. 2025 Data from: Nematode mind: Exploring the role of the RNAi pathway in learning, memory and beyond. Dryad Digital Repository. (10.5061/dryad.12jm63z8r) PMC12198895 40566921

[69] Zwoinska MK, Paida V, Chen H yen, Darkes L, Lind M. 2025 Supplementary material from: Nematode mind: exploring the role of the RNAi pathway in learning, memory and beyond. Figshare. (10.6084/m9.figshare.c.7819729) PMC12198895 40566921

